# (2,7-Dimeth­oxy­naphthalen-1-yl)(3-nitro­phen­yl)methanone

**DOI:** 10.1107/S1600536810042819

**Published:** 2010-10-30

**Authors:** Kotaro Kataoka, Takahiro Nishijima, Atsushi Nagasawa, Akiko Okamoto, Noriyuki Yonezawa

**Affiliations:** a Department of Organic and Polymer Materials Chemistry, Tokyo University of Agriculture & Technology, 2-24-16 Naka-machi, Koganei, Tokyo 184-8588, Japan

## Abstract

The title compound, C_19_H_15_NO_5_, has an intra­molecular C—H⋯O=C hydrogen bond between a naphthalene H atom and the O atom of the carbonyl group. The inter­planar angle between the naphthalene ring system and the benzene ring is 69.59 (5)°. The dihedral angle between the bridging carbonyl C—C(=O)—C plane and the naphthalene ring system is 61.02 (6)°, which is far larger than that between the bridging carbonyl plane and the benzene ring [12.68 (7)°]. The nitro group is slightly out of the plane of the benzene ring [O—N—C—C torsion angle = 4.97 (17)°]. In the crystal, the packing is mainly stabilized by C—H⋯O inter­actions between an H atom of the benzene ring and an O atom of the nitro group.

## Related literature

For the electrophilic aromatic aroylation of 2,7-dimethoxynaphthalene giving aroylated naphthalene compounds, see: Okamoto & Yonezawa (2009 ▶). For the structures of closely related compounds, see: Kato *et al.* (2010 ▶); Mitsui *et al.* (2008 ▶); Muto *et al.* (2010 ▶); Nishijima *et al.* (2010 ▶); Watanabe *et al.* (2010 ▶).
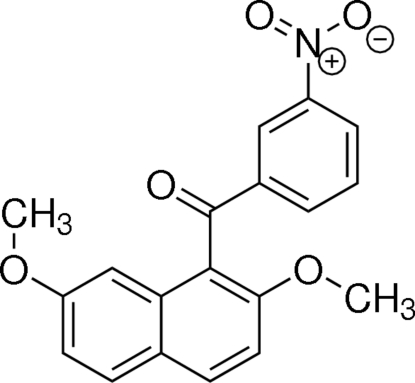


## Experimental

### 

#### Crystal data

C_19_H_15_NO_5_
*M* *_r_* = 337.32
Monoclinic, 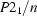

*a* = 8.05658 (18) Å
*b* = 17.0634 (4) Å
*c* = 11.7660 (3) Å
β = 94.660 (1)°
*V* = 1612.15 (6) Å^3^
*Z* = 4
Cu *K*α radiation
μ = 0.85 mm^−1^
*T* = 193 K
0.55 × 0.20 × 0.20 mm

#### Data collection

Rigaku R-AXIS RAPID diffractometer
Absorption correction: numerical (*NUMABS*; Higashi, 1999 ▶) *T*_min_ = 0.653, *T*_max_ = 0.849
29060 measured reflections
2942 independent reflections
2685 reflections with *I* > 2σ(*I*)
*R*_int_ = 0.022

#### Refinement

*R*[*F* ^2^ > 2σ(*F* ^2^)] = 0.033
*wR*(*F* ^2^) = 0.090
*S* = 1.00
2942 reflections
229 parameters
H-atom parameters constrained
Δρ_max_ = 0.19 e Å^−3^
Δρ_min_ = −0.14 e Å^−3^

### 

Data collection: *PROCESS-AUTO* (Rigaku, 1998 ▶); cell refinement: *PROCESS-AUTO*; data reduction: *CrystalStructure* (Rigaku/MSC, 2004 ▶); program(s) used to solve structure: *SIR2004* (Burla *et al.*, 2005 ▶); program(s) used to refine structure: *SHELXL97* (Sheldrick, 2008 ▶); molecular graphics: *ORTEPIII* (Burnett & Johnson, 1996 ▶); software used to prepare material for publication: *SHELXL97*.

## Supplementary Material

Crystal structure: contains datablocks global, I. DOI: 10.1107/S1600536810042819/rn2073sup1.cif

Structure factors: contains datablocks I. DOI: 10.1107/S1600536810042819/rn2073Isup2.hkl

Additional supplementary materials:  crystallographic information; 3D view; checkCIF report

## Figures and Tables

**Table 1:** Hydrogen-bond geometry (Å, °)

*D*—H⋯*A*	*D*—H	H⋯*A*	*D*⋯*A*	*D*—H⋯*A*
C4—H4⋯O1^i^	0.95	2.60	3.3150 (15)	132
C9—H9⋯O1	0.95	2.56	3.0935 (14)	116
C17—H17⋯O5^i^	0.95	2.37	3.2028 (15)	146

Symmetry code: (i) 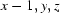.

**Fig. 1.**
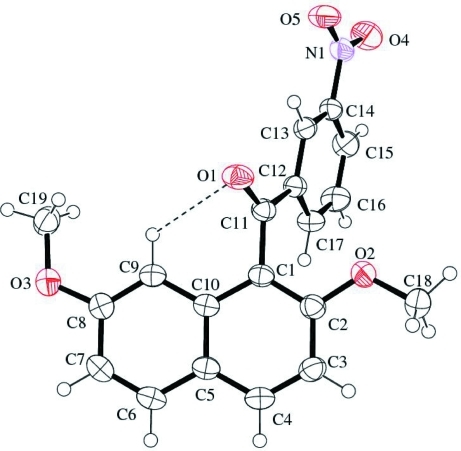
The asymmetric unit of compound (I), showing 50% probability displacement ellipsoids. The dashed line indicates an intramolecular C—H···O hydrogen bond.

**Fig. 2.**
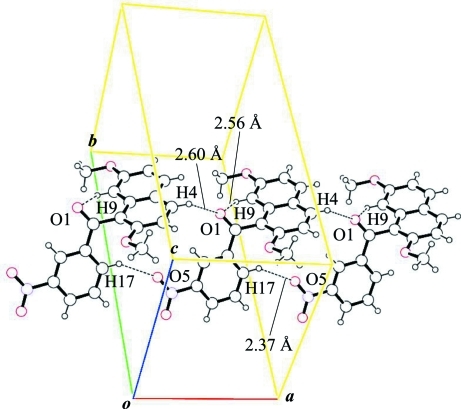
A partial crystal packing diagram of compound (I), viewed down the b axis. The intra- and intermolecular C—H···O hydrogen bonds are shown as dashed lines.

## supplementary crystallographic information

### Comment

In the course of our study on electrophilic aromatic aroylation of 2,7-dimethoxynaphthalene, *peri*-aroylnaphthalene compounds have been found to be formed regioselectively with the aid of suitable acidic mediators (Okamoto & Yonezawa, 2009). We have reported the X-ray crystal structures of 1,8-diaroylated 2,7-dimethoxynaphthalenes such as 1,8-bis(4-methylbenzoyl)-2,7-dimethoxynaphthalene (Muto *et al.*, 2010) and 1,8-bis(4-aminobenzoyl)-2,7-dimethoxynaphthalene (Nishijima *et al.*, 2010). In these compounds, the aroyl groups are oriented in opposite directions. The benzene rings of the aroyl groups are largely out of the plane of the naphthalene ring. Moreover, the ketone carbonyl vectors are out of the planes of the benzene rings and also out of the plane of the naphthalene ring at the same time. The aromatic rings in this type of molecule are assembled with non-coplanar configuration resulting in partial disruption of π-conjugated ring systems. Furthermore, the crystal structures of 1-monoaroylated naphthalene compounds, i. e., 1-(4-chlorobenzoyl)-2,7-dimethoxynaphthalene (Mitsui *et al.*, 2008) and (2,7-dimethoxynaphthalen-1-yl)(phenyl)methanone (Kato *et al.*, 2010), also exhibit essentially the same non-coplanar conformation as the 1,8-diaroylated naphthalene compounds. As a part of our continuous studies on the molecular structures of these kinds of homologous molecules, the X-ray crystal structure of title compound, (I), 1-monoaroylnaphthalene with a nitro substituent, is discussed in this article.

An *ORTEPIII* (Burnett & Johnson, 1996) plot of title compound is displayed in Fig. 1. The interplanar angle between the benzene ring (C12–C17) and the naphthalene ring (C1–C10) is 69.59 (5)°. The bridging carbonyl plane [C1—C11(═O1)—C12] makes dihedral angles with the naphthalene ring system and the benzene ring, *viz.*, 61.02 (6)° [C10—C1—C11—O1 torsion angle = -59.97 (15)°] and 12.68 (7)° [O1—C1—C12—C13 torsion angle = -12.50 (17)°]. The interplanar angle and the dihedral angles are slightly larger than those of 1-(4-nitrobenzoyl)-2,7-dimethoxynaphthalene [Watanabe *et al.*, 2010; interplanar angle = 61.97 (5)°, dihedral angles = 54.68 (6) and 12.54 (7)°]. On the other hand, both 1-monoaroylnaphthalene analogues with a nitro group have a relatively small dihedral angle between the benzene ring and naphthalene ring system compared to other 1-monoaroylnaphthalene homologues. This difference is presumably caused by the intramolecular C—H···O═C interaction, which forms a six-membered ring including the carbonyl group and a naphthalene hydrogen atom (Fig. 1 and Table 1). Besides, the nitro group is slightly out of the plane of the benzene ring [O5—N1—C14—C13 torsion angle = 4.97 (17)°].

In the crystal, the molecular packing is stabilized by C—H···O interactions between a hydrogen atom on the benzene ring and a nitro oxygen atom (C17—H17···O5 = 2.37 Å; Fig. 2 and Table 1). Furthermore, the carbonyl group and the naphthalene ring are connected with a weak C—H···O interaction (C4—H4···O1 = 2.60 Å).

### Experimental

To 50 ml flask, 3-nitrobenzoyl chloride (8.8 mmol, 1.63 g), aluminium chloride (9.7 mmol, 1.29 g) and methylene chloride (10 ml) were placed and stirred at 273 K. To the reaction mixture thus obtained, 2,7-dimethoxynaphthalene (4 mmol, 0.75 g) in methylene chloride (10 ml) were added. After the reaction mixture was stirred at 273 K for 24 h, it was poured into ice-cold water (10 ml). The aqueous layer was extracted with CHCl_3_ (10 ml × 3). The combined extracts were washed with 2 *M* aqueous NaOH followed by washing with brine. The organic layers thus obtained were dried over anhydrous MgSO_4_. The solvent was removed under reduced pressure to give a cake. The crude product was purified by silica gel chromatography (CHCl_3_). Yellow platelet single crystals suitable for X-ray diffraction were obtained by crystallization from hexane and chloroform (45% yield).

Spectroscopic Data: ^1^H NMR (300 MHz, CDCl_3_) δ 3.77 [3.766](3*H*, s), 3.77 [3.772] (3*H*, s), 6.87 (1*H*, d, *J* = 2 Hz), 7.06 (1*H*, dd, *J* = 2, 9 Hz), 7.20 (1*H*, d, *J* = 9 Hz), 7.64 (1*H*, t, *J* = 8 Hz), 7.76 (1*H*, d, *J* = 9 Hz), 7.94 (1*H*, d, *J* = 9 Hz), 8.17 (1*H*, ddd, *J* = 1, 2, 8 Hz), 7.92 (1*H*, ddd, *J* = 1, 2, 8 Hz), 8.65 (1*H*, dd, *J* = 1, 2 Hz) p.p.m..

^13^C NMR (75 MHz, CDCl_3_) δ 55.2, 56.1, 101.7, 109.8, 117.3, 119.7, 124.1, 124.5, 127.4, 129.6, 130.0, 132.3, 133.0, 134.9, 139.7, 148.5, 155.6, 159.3, 195.7 p.p.m..

IR (KBr): 1670, 1624, 1513, 1253 cm^-1^.

Anal. Calcd for C_19_H_15_NO_5_: C, 67.65%; H, 4.48%; Found: C, 67.79%; H, 4.58%.

### Refinement

All the H-atoms could be located in difference Fourier maps. The H atoms attached to carbon were introduced in calculated positions and treated as riding on their parent atoms with C—H = 0.98 Å (methyl) or 0.95 Å (aromatic) with *U*_iso_(H) = 1.2*U*_eq_(C_aromatic_) or *U*_iso_(H) = 1.5*U*_eq_(C_methyl_).

### Crystal data

**Table:**

C_19_H_15_NO_5_	*F*(000) = 704
*M**_r_* = 337.32	*D*_x_ = 1.390 Mg m^−^^3^
Monoclinic, *P*2_1_/*n*	Melting point = 418.8–419.1 K
Hall symbol: -P 2yn	Cu *K*α radiation, λ = 1.54187 Å
*a* = 8.05658 (18) Å	Cell parameters from 23523 reflections
*b* = 17.0634 (4) Å	θ = 3.8–68.2°
*c* = 11.7660 (3) Å	µ = 0.85 mm^−^^1^
β = 94.660 (1)°	*T* = 193 K
*V* = 1612.15 (6) Å^3^	Block, yellow
*Z* = 4	0.55 × 0.20 × 0.20 mm

### Data collection

**Table:**

Rigaku R-AXIS RAPID diffractometer	2942 independent reflections
Radiation source: rotating anode	2685 reflections with *I* > 2σ(*I*)
graphite	*R*_int_ = 0.022
Detector resolution: 10.00 pixels mm^-1^	θ_max_ = 68.2°, θ_min_ = 4.6°
ω scans	*h* = −9→9
Absorption correction: numerical (*NUMABS*; Higashi, 1999)	*k* = −20→20
*T*_min_ = 0.653, *T*_max_ = 0.849	*l* = −14→14
29060 measured reflections	

### Refinement

**Table:**

Refinement on *F*^2^	Secondary atom site location: difference Fourier map
Least-squares matrix: full	Hydrogen site location: inferred from neighbouring sites
*R*[*F*^2^ > 2σ(*F*^2^)] = 0.033	H-atom parameters constrained
*wR*(*F*^2^) = 0.090	*w* = 1/[σ^2^(*F*_o_^2^) + (0.0474*P*)^2^ + 0.4684*P*] where *P* = (*F*_o_^2^ + 2*F*_c_^2^)/3
*S* = 1.00	(Δ/σ)_max_ < 0.001
2942 reflections	Δρ_max_ = 0.19 e Å^−^^3^
229 parameters	Δρ_min_ = −0.14 e Å^−^^3^
0 restraints	Extinction correction: *SHELXL97* (Sheldrick, 2008), Fc^*^=kFc[1+0.001xFc^2^λ^3^/sin(2θ)]^-1/4^
Primary atom site location: structure-invariant direct methods	Extinction coefficient: 0.0073 (4)

### Special details

**Table:**

Geometry. All e.s.d.'s (except the e.s.d. in the dihedral angle between two l.s. planes) are estimated using the full covariance matrix. The cell e.s.d.'s are taken into account individually in the estimation of e.s.d.'s in distances, angles and torsion angles; correlations between e.s.d.'s in cell parameters are only used when they are defined by crystal symmetry. An approximate (isotropic) treatment of cell e.s.d.'s is used for estimating e.s.d.'s involving l.s. planes.
Refinement. Refinement of *F*^2^ against ALL reflections. The weighted *R*-factor *wR* and goodness of fit *S* are based on *F*^2^, conventional *R*-factors *R* are based on *F*, with *F* set to zero for negative *F*^2^. The threshold expression of *F*^2^ > σ(*F*^2^) is used only for calculating *R*-factors(gt) *etc*. and is not relevant to the choice of reflections for refinement. *R*-factors based on *F*^2^ are statistically about twice as large as those based on *F*, and *R*- factors based on ALL data will be even larger.

### Fractional atomic coordinates and isotropic or equivalent isotropic displacement parameters (Å^2^)

**Table:**

	*x*	*y*	*z*	*U*_iso_*/*U*_eq_	
O1	0.27637 (10)	0.43017 (5)	0.69042 (8)	0.0421 (2)	
O2	0.00689 (11)	0.59531 (5)	0.62855 (8)	0.0451 (3)	
O3	−0.04611 (11)	0.18744 (5)	0.79381 (8)	0.0432 (2)	
O4	0.64787 (14)	0.65633 (7)	1.04665 (10)	0.0638 (3)	
O5	0.71205 (11)	0.57358 (6)	0.91928 (9)	0.0526 (3)	
N1	0.61035 (14)	0.60860 (6)	0.97139 (10)	0.0416 (3)	
C1	−0.00844 (14)	0.46560 (7)	0.68859 (9)	0.0309 (3)	
C2	−0.08844 (15)	0.52991 (7)	0.63812 (10)	0.0343 (3)	
C3	−0.25834 (16)	0.52651 (8)	0.59896 (10)	0.0377 (3)	
H3	−0.3127	0.5713	0.5655	0.045*	
C4	−0.34356 (15)	0.45847 (8)	0.60951 (10)	0.0371 (3)	
H4	−0.4586	0.4568	0.5843	0.044*	
C5	−0.26677 (14)	0.39048 (7)	0.65650 (9)	0.0328 (3)	
C6	−0.35374 (15)	0.31846 (8)	0.66192 (10)	0.0372 (3)	
H6	−0.4679	0.3161	0.6347	0.045*	
C7	−0.27714 (16)	0.25281 (8)	0.70512 (10)	0.0386 (3)	
H7	−0.3364	0.2047	0.7061	0.046*	
C8	−0.10780 (15)	0.25655 (7)	0.74891 (10)	0.0342 (3)	
C9	−0.01892 (14)	0.32474 (7)	0.74566 (9)	0.0318 (3)	
H9	0.0942	0.3262	0.7755	0.038*	
C10	−0.09579 (14)	0.39342 (7)	0.69773 (9)	0.0298 (3)	
C11	0.17233 (14)	0.47124 (7)	0.73012 (9)	0.0307 (3)	
C12	0.22134 (14)	0.52681 (7)	0.82550 (9)	0.0298 (3)	
C13	0.38964 (14)	0.54220 (7)	0.85296 (10)	0.0311 (3)	
H13	0.4725	0.5190	0.8108	0.037*	
C14	0.43276 (15)	0.59185 (7)	0.94285 (10)	0.0341 (3)	
C15	0.31664 (17)	0.62608 (8)	1.00789 (11)	0.0425 (3)	
H15	0.3505	0.6597	1.0699	0.051*	
C16	0.15028 (17)	0.61002 (8)	0.98007 (11)	0.0444 (3)	
H16	0.0681	0.6327	1.0234	0.053*	
C17	0.10273 (15)	0.56103 (8)	0.88928 (10)	0.0368 (3)	
H17	−0.0121	0.5507	0.8704	0.044*	
C18	−0.05995 (17)	0.65802 (8)	0.55877 (11)	0.0412 (3)	
H18A	0.0266	0.6975	0.5507	0.049*	
H18B	−0.1535	0.6819	0.5943	0.049*	
H18C	−0.0988	0.6377	0.4834	0.049*	
C19	0.12202 (18)	0.18734 (8)	0.84244 (12)	0.0461 (3)	
H19A	0.1498	0.1354	0.8740	0.055*	
H19B	0.1349	0.2266	0.9033	0.055*	
H19C	0.1968	0.2000	0.7834	0.055*	

### Atomic displacement parameters (Å^2^)

**Table:**

	*U*^11^	*U*^22^	*U*^33^	*U*^12^	*U*^13^	*U*^23^
O1	0.0304 (5)	0.0488 (5)	0.0476 (5)	0.0002 (4)	0.0063 (4)	−0.0138 (4)
O2	0.0411 (5)	0.0371 (5)	0.0550 (6)	−0.0072 (4)	−0.0099 (4)	0.0087 (4)
O3	0.0441 (5)	0.0346 (5)	0.0504 (5)	−0.0017 (4)	−0.0003 (4)	0.0029 (4)
O4	0.0542 (7)	0.0562 (6)	0.0767 (7)	−0.0120 (5)	−0.0224 (6)	−0.0144 (6)
O5	0.0293 (5)	0.0639 (7)	0.0639 (6)	−0.0023 (4)	−0.0007 (4)	0.0040 (5)
N1	0.0356 (6)	0.0387 (6)	0.0484 (6)	−0.0068 (5)	−0.0087 (5)	0.0058 (5)
C1	0.0280 (6)	0.0360 (6)	0.0287 (5)	−0.0012 (5)	0.0016 (4)	−0.0036 (5)
C2	0.0340 (6)	0.0349 (6)	0.0338 (6)	−0.0023 (5)	0.0002 (5)	−0.0021 (5)
C3	0.0346 (7)	0.0406 (7)	0.0370 (6)	0.0039 (5)	−0.0038 (5)	0.0010 (5)
C4	0.0272 (6)	0.0481 (7)	0.0351 (6)	0.0002 (5)	−0.0023 (5)	−0.0016 (5)
C5	0.0288 (6)	0.0421 (7)	0.0275 (6)	−0.0024 (5)	0.0024 (4)	−0.0034 (5)
C6	0.0287 (6)	0.0476 (7)	0.0350 (6)	−0.0078 (5)	0.0004 (5)	−0.0025 (5)
C7	0.0391 (7)	0.0401 (7)	0.0369 (6)	−0.0109 (5)	0.0048 (5)	−0.0031 (5)
C8	0.0378 (6)	0.0348 (6)	0.0302 (6)	−0.0010 (5)	0.0049 (5)	−0.0021 (5)
C9	0.0277 (6)	0.0387 (6)	0.0289 (5)	0.0000 (5)	0.0019 (4)	−0.0030 (5)
C10	0.0280 (6)	0.0365 (6)	0.0252 (5)	−0.0016 (5)	0.0033 (4)	−0.0039 (4)
C11	0.0280 (6)	0.0334 (6)	0.0310 (6)	−0.0014 (5)	0.0042 (5)	0.0014 (5)
C12	0.0271 (6)	0.0324 (6)	0.0300 (6)	−0.0009 (4)	0.0018 (4)	0.0017 (4)
C13	0.0283 (6)	0.0324 (6)	0.0326 (6)	0.0014 (5)	0.0029 (5)	0.0033 (5)
C14	0.0305 (6)	0.0338 (6)	0.0368 (6)	−0.0036 (5)	−0.0042 (5)	0.0042 (5)
C15	0.0461 (8)	0.0417 (7)	0.0386 (7)	−0.0012 (6)	−0.0023 (6)	−0.0089 (5)
C16	0.0395 (7)	0.0521 (8)	0.0422 (7)	0.0048 (6)	0.0075 (6)	−0.0115 (6)
C17	0.0273 (6)	0.0455 (7)	0.0377 (6)	0.0006 (5)	0.0030 (5)	−0.0034 (5)
C18	0.0481 (8)	0.0356 (6)	0.0396 (7)	0.0013 (6)	0.0021 (6)	0.0020 (5)
C19	0.0496 (8)	0.0389 (7)	0.0482 (8)	0.0051 (6)	−0.0053 (6)	0.0003 (6)

### Geometric parameters (Å, °)

**Table:**

O1—C11	1.2147 (14)	C7—H7	0.9500
O2—C2	1.3646 (15)	C8—C9	1.3685 (17)
O2—C18	1.4273 (15)	C9—C10	1.4206 (17)
O3—C8	1.3689 (15)	C9—H9	0.9500
O3—C19	1.4270 (16)	C11—C12	1.4974 (16)
O4—N1	1.2229 (15)	C12—C17	1.3906 (16)
O5—N1	1.2189 (15)	C12—C13	1.3931 (16)
N1—C14	1.4709 (16)	C13—C14	1.3774 (17)
C1—C2	1.3821 (17)	C13—H13	0.9500
C1—C10	1.4269 (16)	C14—C15	1.3851 (18)
C1—C11	1.5012 (16)	C15—C16	1.3810 (19)
C2—C3	1.4096 (17)	C15—H15	0.9500
C3—C4	1.3596 (18)	C16—C17	1.3858 (18)
C3—H3	0.9500	C16—H16	0.9500
C4—C5	1.4067 (18)	C17—H17	0.9500
C4—H4	0.9500	C18—H18A	0.9800
C5—C6	1.4187 (17)	C18—H18B	0.9800
C5—C10	1.4236 (16)	C18—H18C	0.9800
C6—C7	1.3576 (18)	C19—H19A	0.9800
C6—H6	0.9500	C19—H19B	0.9800
C7—C8	1.4201 (18)	C19—H19C	0.9800
			
C2—O2—C18	118.16 (10)	C5—C10—C1	118.31 (11)
C8—O3—C19	117.31 (10)	O1—C11—C12	120.36 (10)
O5—N1—O4	123.64 (11)	O1—C11—C1	121.29 (10)
O5—N1—C14	118.11 (11)	C12—C11—C1	118.31 (10)
O4—N1—C14	118.25 (12)	C17—C12—C13	119.62 (11)
C2—C1—C10	120.14 (11)	C17—C12—C11	121.29 (10)
C2—C1—C11	119.68 (10)	C13—C12—C11	119.05 (10)
C10—C1—C11	120.15 (10)	C14—C13—C12	118.30 (11)
O2—C2—C1	116.03 (10)	C14—C13—H13	120.9
O2—C2—C3	123.01 (11)	C12—C13—H13	120.9
C1—C2—C3	120.96 (11)	C13—C14—C15	122.96 (11)
C4—C3—C2	119.31 (12)	C13—C14—N1	118.34 (11)
C4—C3—H3	120.3	C15—C14—N1	118.69 (11)
C2—C3—H3	120.3	C16—C15—C14	118.13 (11)
C3—C4—C5	122.06 (11)	C16—C15—H15	120.9
C3—C4—H4	119.0	C14—C15—H15	120.9
C5—C4—H4	119.0	C15—C16—C17	120.31 (12)
C4—C5—C6	121.94 (11)	C15—C16—H16	119.8
C4—C5—C10	119.17 (11)	C17—C16—H16	119.8
C6—C5—C10	118.88 (11)	C16—C17—C12	120.68 (11)
C7—C6—C5	121.36 (11)	C16—C17—H17	119.7
C7—C6—H6	119.3	C12—C17—H17	119.7
C5—C6—H6	119.3	O2—C18—H18A	109.5
C6—C7—C8	119.53 (11)	O2—C18—H18B	109.5
C6—C7—H7	120.2	H18A—C18—H18B	109.5
C8—C7—H7	120.2	O2—C18—H18C	109.5
C9—C8—O3	124.67 (11)	H18A—C18—H18C	109.5
C9—C8—C7	121.17 (11)	H18B—C18—H18C	109.5
O3—C8—C7	114.16 (11)	O3—C19—H19A	109.5
C8—C9—C10	120.02 (11)	O3—C19—H19B	109.5
C8—C9—H9	120.0	H19A—C19—H19B	109.5
C10—C9—H9	120.0	O3—C19—H19C	109.5
C9—C10—C5	119.00 (11)	H19A—C19—H19C	109.5
C9—C10—C1	122.69 (10)	H19B—C19—H19C	109.5
			
C18—O2—C2—C1	168.50 (11)	C2—C1—C10—C9	178.10 (11)
C18—O2—C2—C3	−11.15 (17)	C11—C1—C10—C9	0.20 (16)
C10—C1—C2—O2	−177.16 (10)	C2—C1—C10—C5	−1.63 (16)
C11—C1—C2—O2	0.74 (16)	C11—C1—C10—C5	−179.53 (10)
C10—C1—C2—C3	2.49 (18)	C2—C1—C11—O1	−117.94 (13)
C11—C1—C2—C3	−179.60 (10)	C10—C1—C11—O1	59.96 (15)
O2—C2—C3—C4	178.56 (11)	C2—C1—C11—C12	64.42 (14)
C1—C2—C3—C4	−1.07 (18)	C10—C1—C11—C12	−117.67 (12)
C2—C3—C4—C5	−1.23 (19)	O1—C11—C12—C17	−165.28 (11)
C3—C4—C5—C6	−176.62 (11)	C1—C11—C12—C17	12.37 (16)
C3—C4—C5—C10	2.03 (18)	O1—C11—C12—C13	12.50 (17)
C4—C5—C6—C7	178.48 (11)	C1—C11—C12—C13	−169.84 (10)
C10—C5—C6—C7	−0.17 (18)	C17—C12—C13—C14	−0.50 (17)
C5—C6—C7—C8	1.83 (18)	C11—C12—C13—C14	−178.32 (10)
C19—O3—C8—C9	1.23 (17)	C12—C13—C14—C15	0.96 (18)
C19—O3—C8—C7	−178.24 (11)	C12—C13—C14—N1	−179.56 (10)
C6—C7—C8—C9	−1.73 (18)	O5—N1—C14—C13	−4.97 (17)
C6—C7—C8—O3	177.76 (11)	O4—N1—C14—C13	175.63 (11)
O3—C8—C9—C10	−179.52 (10)	O5—N1—C14—C15	174.53 (12)
C7—C8—C9—C10	−0.09 (17)	O4—N1—C14—C15	−4.87 (17)
C8—C9—C10—C5	1.75 (16)	C13—C14—C15—C16	−0.7 (2)
C8—C9—C10—C1	−177.99 (10)	N1—C14—C15—C16	179.87 (12)
C4—C5—C10—C9	179.68 (10)	C14—C15—C16—C17	−0.1 (2)
C6—C5—C10—C9	−1.63 (16)	C15—C16—C17—C12	0.5 (2)
C4—C5—C10—C1	−0.57 (16)	C13—C12—C17—C16	−0.24 (19)
C6—C5—C10—C1	178.12 (10)	C11—C12—C17—C16	177.54 (12)

### Hydrogen-bond geometry (Å, °)

**Table:**

*D*—H···*A*	*D*—H	H···*A*	*D*···*A*	*D*—H···*A*
C4—H4···O1^i^	0.95	2.60	3.3150 (15)	132
C9—H9···O1	0.95	2.56	3.0935 (14)	116
C17—H17···O5^i^	0.95	2.37	3.2028 (15)	146

Symmetry codes: (i) *x*−1, *y*, *z*.
